# Supplementary material for: Innovation and elaboration on the avian tree of life
Source: Sci Adv. 2023 Oct 25;9(43):eadg1641. doi: 10.1126/sciadv.adg1641 (PMC10599619; doi:10.1126/sciadv.adg1641)
Supplement: Supplementary file 1 — Supplementary Text Figs. S1 to S18 Table S1 References [file sciadv.adg1641_sm.pdf]

Science Advances
AAAS

Supplementary Materials for

Innovation and elaboration on the avian tree of life

Thomas Guillerme *et al.*

Corresponding author: Thomas Guillerme, guillert@tcd.ie

Sci. Adv. **9**, eadg1641 (2023)
DOI: 10.1126/sciadv.adg1641

This PDF file includes:

Supplementary Text
Figs. S1 to S18
Table S1
References

1 Additional methods details

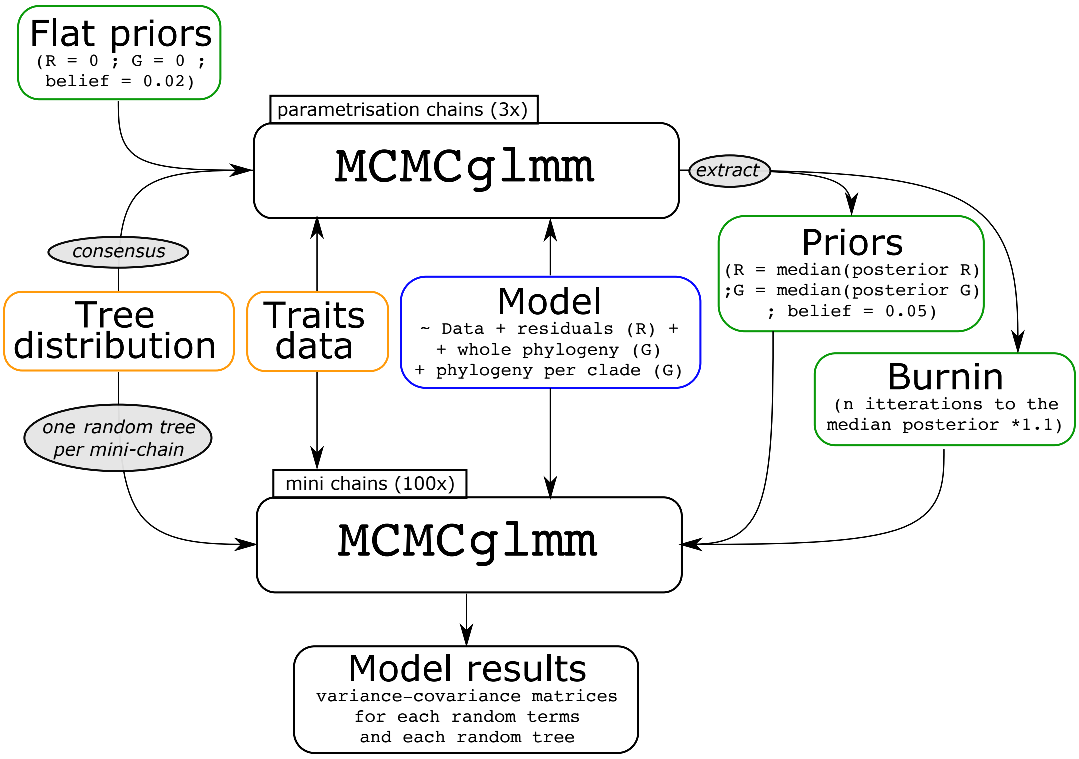

Figure S1: mcmcmcglmmm: mini-chains MCMCglmm method diagram. See Methods for more details.

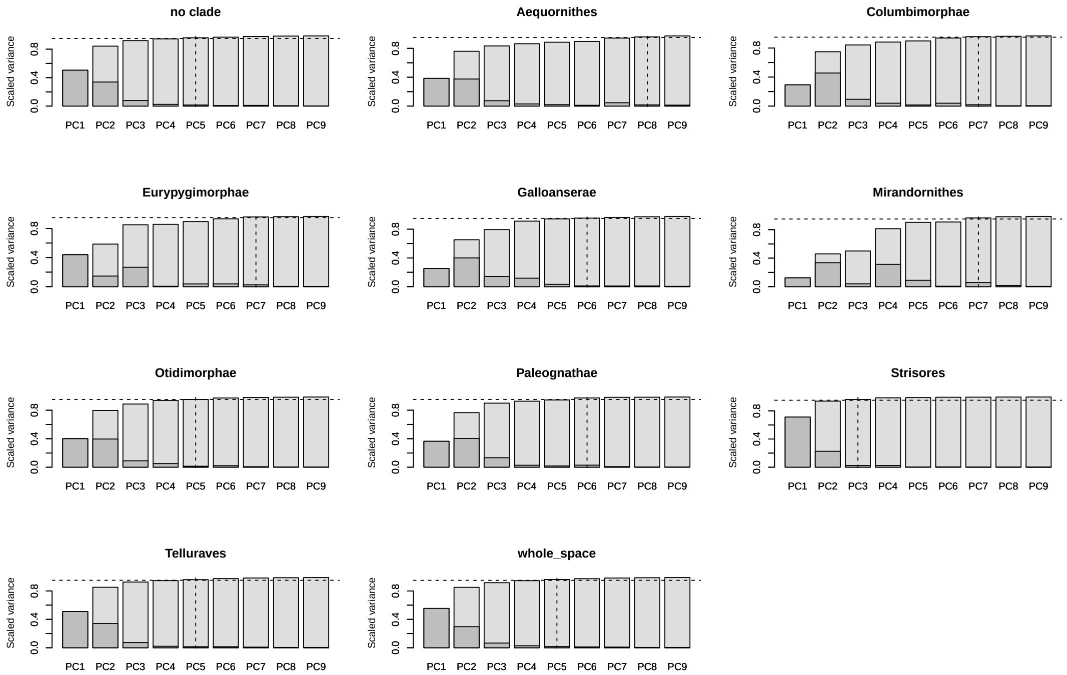

Figure S2: Variance and cumulative variance for each of the eight principal component (PC) axes for each super-order. Note that not all super-orders have their own variance explained matching the variance explained by the entire ordination. For example, Strisores only need the first three dimensions to explain at least 95% of their variance within the shapespace whilst Aequornithes need all eight dimensions to achieve at least the same amount of variance explained. *noclade* is the cumulative variance for species not present in the other displayed clades and *whole_space* is the cumulative for all species.

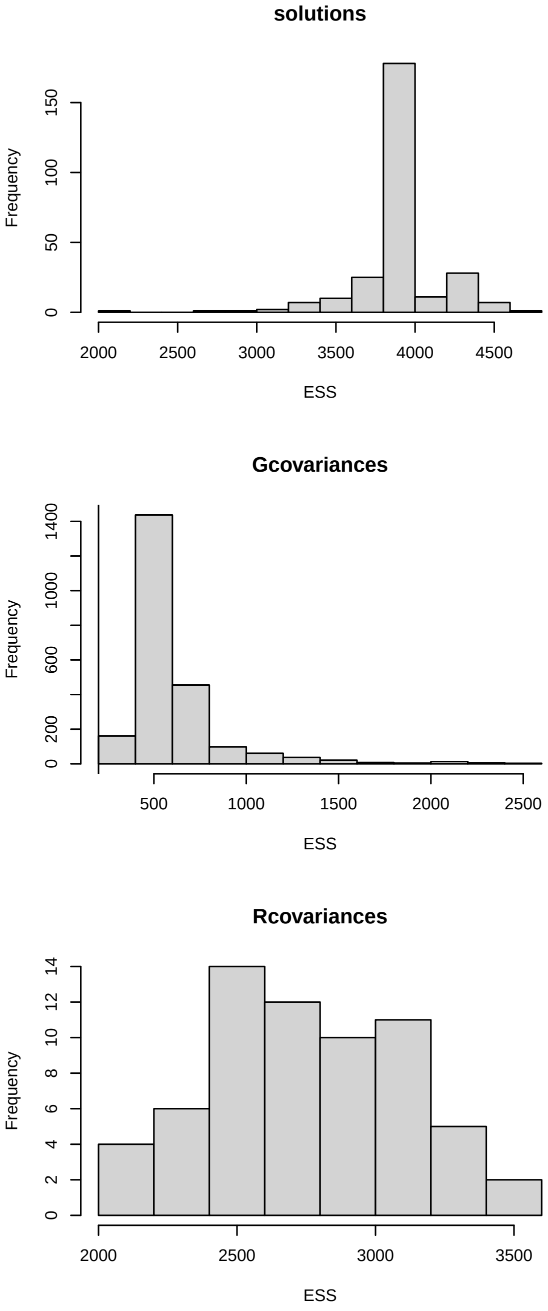

Figure S3: Histogram of the effective sample sizes (ESS) for each class of parameters in the MCMCglmm model for all birds for the 4000 posteriors. **solutions** is the solution of the model; **Gcovariances** is the covariance for the random terms (the phylogeny and the clades); **Rcovariances** is the residual error term.

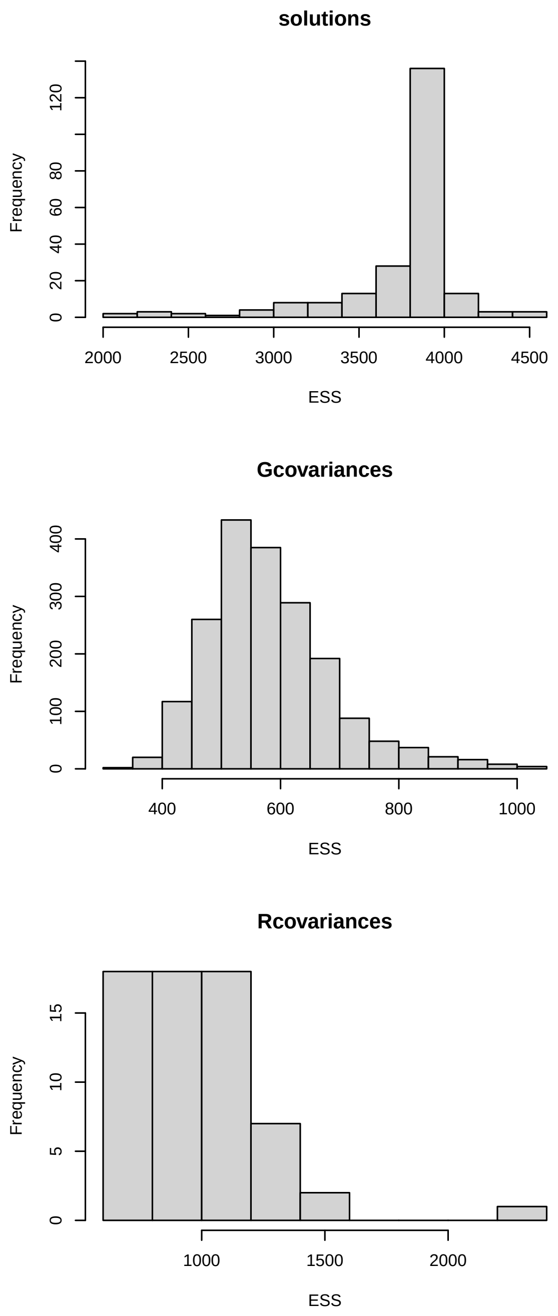

Figure S4: Histogram of the effective sample sizes (ESS) for each class of parameters in the MCMCglmm model for the Passeriformes for the 4000 posteriors. **solutions** is the solution of the model; **Gcovariances** is the covariance for the random terms (the phylogeny and the clades); **Rcovariances** is the residual error term.

2 Additional results

2.1 Elaboration and innovation at the super-order or clade-wide phylogenetic level

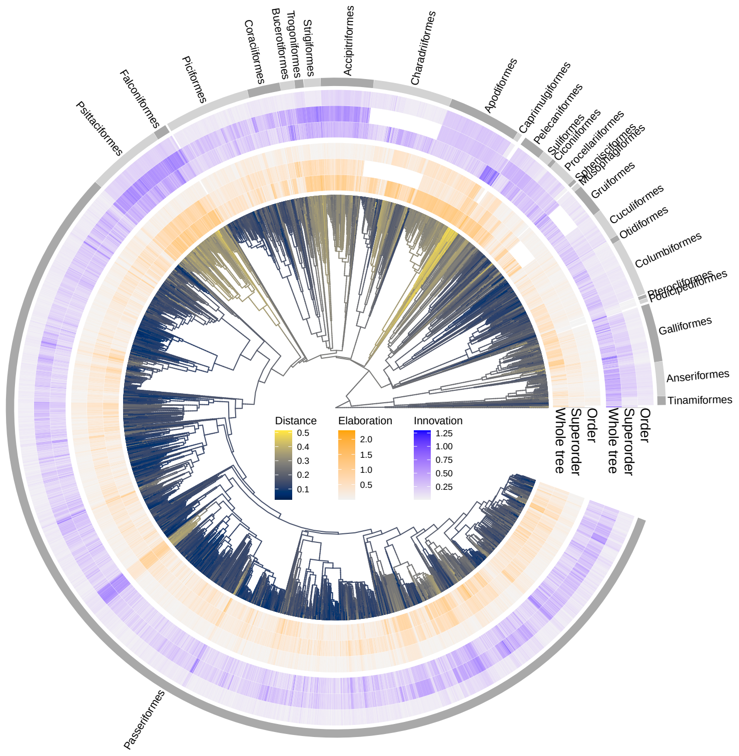

Figure S5: Avian phylogeny (n = 8748 species) showing Euclidean distance of species to the centroid of beak space (branches, cividis scale), and distributions of species beak shape $elaboration_{species}$ (inner circle, orange scale), and $innovation_{species}$ (outer circle, blue scale). Elaboration and innovation scores represent comparisons of species at the order, super-order and class-wide phylogenetic level.

2.2 Correlations between $elaboration_{species}$ and $innovation_{species}$

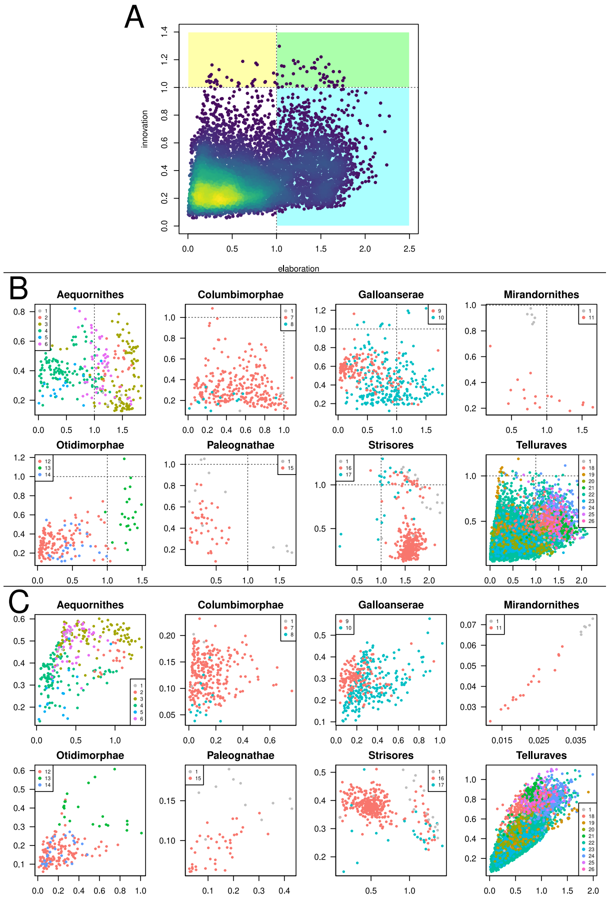

Figure S6: Median correlation plots of $elaboration_{species}$ and $innovation_{species}$ at different scales. **(A)** Correlation between $elaboration_{species}$ and $innovation_{species}$ for each species when projected on the whole phylogeny. The colours of the dots correspond to their density in the plot. Blue and yellow colours corresponds to respectively low and high density of species. The coloured quadrant in the background corresponds to $elaboration_{species}$ and $innovation_{species}$ scores higher than 1. The blue and yellow quadrants shows species with noticeably high $elaboration_{species}$ and $innovation_{species}$ respectively. The species in the green quadrant have noticeably high levels of both $elaboration_{species}$ and $innovation_{species}$. **(B)** Correlation between $elaboration_{species}$ and $innovation_{species}$ for each species when projected on the whole phylogeny (mega-level projection) but plotted by super-order and coloured by order (see list below). **C** correlation between $elaboration_{species}$ and $innovation_{species}$ for each species when projected on their super-orders' major axis of variation (macro-level projection). The colours correspond to each order. For the distribution of correlation scores, see Fig. 3. The group numbers are: 1) others (groups with < 15 species) 2) Corvoidea; 3) Malaconotoidea; 4) Orioloidea; 5) Meliphagoidea; 6) Bombycilloidea; 7) Muscicapoidea; 8) Emberizoidea; 9) Motacillidae; 10) Nectariniidae; 11) Passeridae; 12) PloceidaeEstrildidae; 13) Eurylaimides; 14) Furnariida; 15) Tyrannida; 16) Aegithaloidea; 17) Cisticolidae; 18) Fringillidae; 19) Hirundinidae; 20) Locustelloidea; 21) Paridae; 22) Pycnonotidae; 23) Sylvioidea.

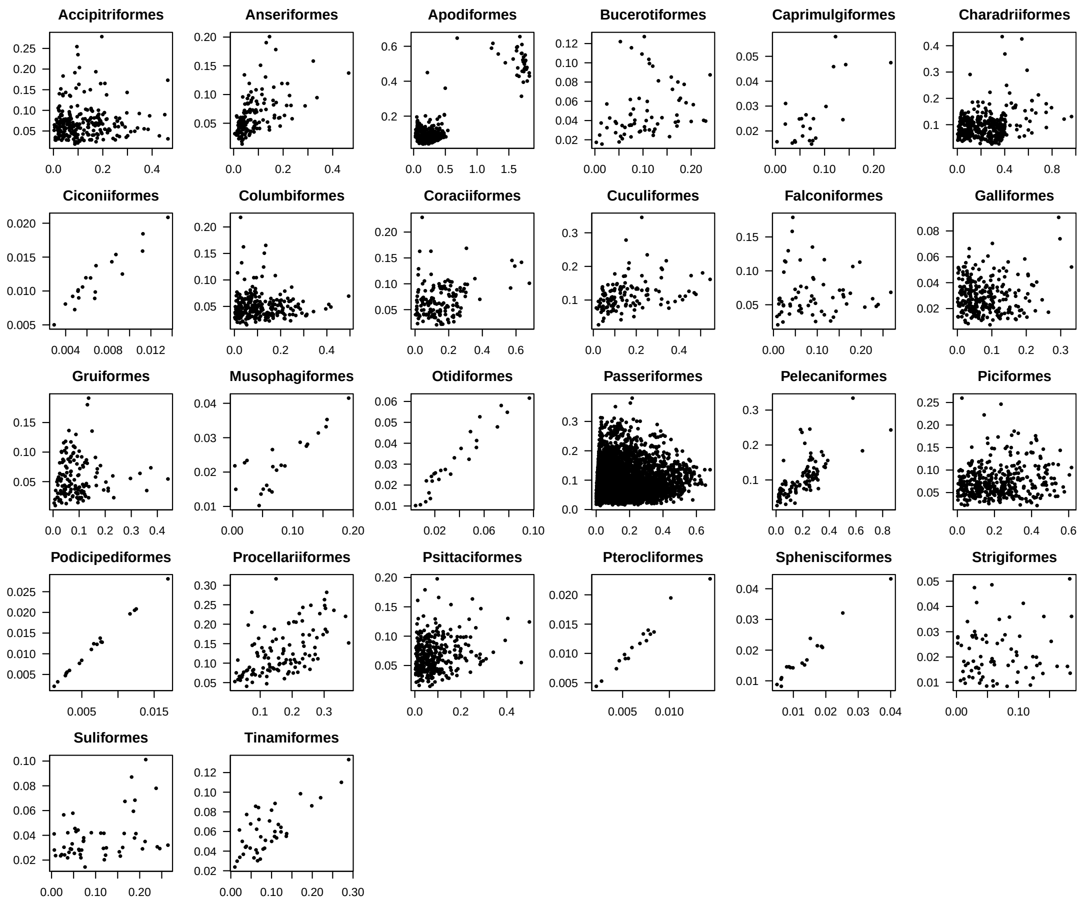

Figure S7: Median correlation plots of $elaboration_{species}$ and $innovation_{species}$ for each species projected on their order's phylogenetic major axis of beak variation. Each point is the median $elaboration_{species}$ and $innovation_{species}$ for one species from the distribution of 4000 posterior evolutionary rate matrices. The full distribution of the correlations between $elaboration_{species}$ and $innovation_{species}$ is available in the main text in Figure 3.

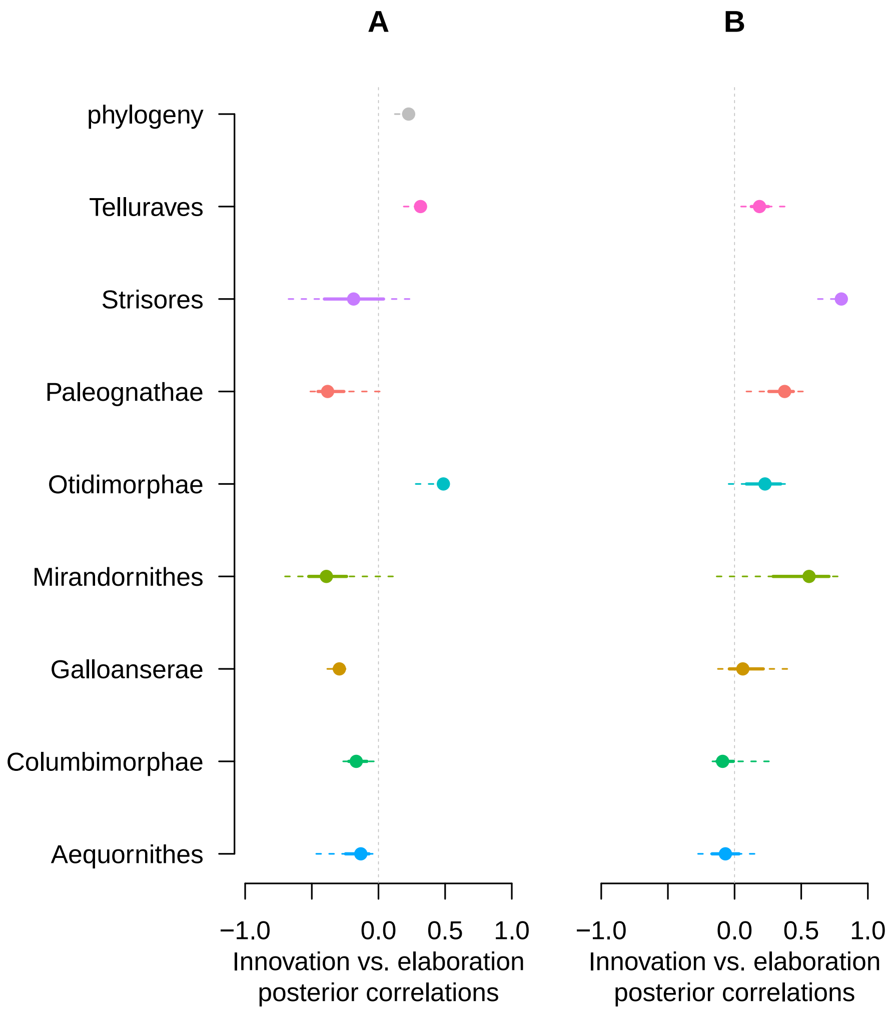

Figure S8: Posterior distributions of the correlation between $elaboration_{species}$ and $innovation_{species}$ for each super-orders in Fig. S6. A) Species' projection onto the class-wide phylogenetic major axes of beak variation (corresponding to the correlation scores in Fig. S6A-B); B) Species' projection onto the super-orders' phylogenetic major axes of beak variation (corresponding to the correlation scores in Fig. S6C).

Table S1: Posterior variance-covariance ellipses results for each clade compared to their parent clade or their parent's parent's clade (Comparison). n = the number of species in each group. sd = the standard deviation of the ellipses orientation (across the posterior distribution). orthogonality = the degree of right angle for each group compared to their parent or parent's group (0 = parallel, 1 = orthogonal). Post. prob = the posterior probability of the orthogonality in the focal group being different from the comparison one (the 95% CI is from the randomised posterior probabilities).

Group	Comparison	n	ellipse sd	orthogonality	2.5%	97.5%	Post. prob.	2.5%	97.5%
Aequornithes	phylogeny	308	15.465	0.710	0.219	0.988	0.807	0.802	0.817
Columbimorphae	phylogeny	285	5.916	0.884	0.564	0.995	0.999	0.996	0.999
Galloanserae	phylogeny	421	13.489	0.800	0.369	0.992	0.823	0.814	0.829
Mirandornithes	phylogeny	24	20.870	0.839	0.448	0.992	0.490	0.486	0.503
Otidimorphae	phylogeny	181	16.800	0.680	0.291	0.983	0.803	0.796	0.813
Paleognathae	phylogeny	52	20.728	0.718	0.264	0.985	0.804	0.800	0.815
Strisores	phylogeny	370	16.237	0.787	0.469	0.986	0.994	0.991	0.995
Telluraves	phylogeny	6604	14.339	0.730	0.294	0.988	0.657	0.643	0.659
Accipitriformes	phylogeny	240	5.044	0.906	0.706	0.996	1.000	1.000	1.000
Anseriformes	phylogeny	156	13.546	0.743	0.167	0.989	0.768	0.768	0.782
Apodiformes	phylogeny	331	18.307	0.684	0.326	0.971	0.891	0.882	0.895
Bucerotiformes	phylogeny	66	4.651	0.857	0.542	0.993	0.984	0.981	0.986
Caprimulgiformes	phylogeny	23	11.555	0.847	0.533	0.992	0.941	0.935	0.943
Charadriiformes	phylogeny	359	5.632	0.331	0.148	0.592	0.923	0.915	0.925
Ciconiiformes	phylogeny	19	20.915	0.839	0.459	0.992	0.523	0.509	0.529
Columbiformes	phylogeny	266	3.975	0.896	0.649	0.996	1.000	1.000	1.000
Coraciiformes	phylogeny	149	17.618	0.358	0.196	0.603	0.980	0.976	0.983
Cuculiformes	phylogeny	134	18.927	0.603	0.217	0.970	0.744	0.742	0.759
Falconiformes	phylogeny	63	12.521	0.851	0.520	0.993	0.978	0.976	0.982
Galliformes	phylogeny	265	1.416	0.894	0.694	0.995	1.000	1.000	1.000
Gruiformes	phylogeny	138	7.922	0.543	0.215	0.894	0.942	0.939	0.951
Musophagiformes	phylogeny	22	11.601	0.898	0.581	0.996	0.924	0.918	0.928
Otidiformes	phylogeny	25	17.148	0.706	0.271	0.983	0.481	0.473	0.491
Passeriformes	phylogeny	5229	11.642	0.428	0.189	0.650	0.999	0.999	1.000

Pelecaniformes	phylogeny	103	28.435	0.537	0.284	0.937	0.752	0.750	0.763
Piciformes	phylogeny	390	15.366	0.460	0.216	0.684	0.999	0.998	1.000
Podicipediformes	phylogeny	18	20.335	0.828	0.453	0.993	0.493	0.480	0.501
Procellariiformes	phylogeny	112	13.408	0.529	0.241	0.949	0.619	0.602	0.618
Psittaciformes	phylogeny	341	12.975	0.572	0.433	0.755	1.000	1.000	1.000
Pterocliformes	phylogeny	16	21.382	0.834	0.459	0.994	0.504	0.498	0.513
Sphenisciformes	phylogeny	17	21.149	0.809	0.415	0.991	0.493	0.490	0.508
Strigiformes	phylogeny	76	2.241	0.921	0.721	0.996	1.000	1.000	1.000
Suliformes	phylogeny	52	7.537	0.745	0.437	0.976	0.995	0.992	0.995
Tinamiformes	phylogeny	41	18.518	0.805	0.331	0.991	0.714	0.708	0.723
Trogoniformes	phylogeny	41	9.463	0.882	0.593	0.995	0.996	0.995	0.998
Procellariiformes	Aequornithes	112	13.408	0.677	0.263	0.986	0.690	0.686	0.702
Sphenisciformes	Aequornithes	17	21.149	0.744	0.348	0.988	0.406	0.405	0.421
Ciconiiformes	Aequornithes	19	20.915	0.790	0.365	0.987	0.442	0.429	0.448
Pelecaniformes	Aequornithes	103	28.435	0.541	0.196	0.968	0.662	0.657	0.673
Suliformes	Aequornithes	52	7.537	0.299	0.128	0.875	0.767	0.755	0.773
Columbiformes	Columbimorphae	266	3.975	0.182	0.079	0.424	0.634	0.622	0.636
Pterocliformes	Columbimorphae	16	21.382	0.801	0.396	0.993	0.469	0.449	0.467
Galliformes	Galloanserae	265	1.416	0.327	0.146	0.905	1.000	1.000	1.000
Anseriformes	Galloanserae	156	13.546	0.499	0.206	0.963	0.604	0.595	0.610
Podicipediformes	Mirandornithes	18	20.335	0.552	0.170	0.972	0.489	0.492	0.505
Otidiformes	Otidimorphae	25	17.148	0.608	0.208	0.980	0.414	0.405	0.423
Cuculiformes	Otidimorphae	134	18.927	0.486	0.173	0.959	0.525	0.521	0.536
Musophagiformes	Otidimorphae	22	11.601	0.487	0.181	0.963	0.588	0.587	0.603
Tinamiformes	Paleognathae	41	18.518	0.455	0.145	0.964	0.432	0.427	0.445
Caprimulgiformes	Strisores	23	11.555	0.327	0.152	0.858	0.513	0.505	0.522
Apodiformes	Strisores	331	18.307	0.340	0.141	0.797	0.521	0.514	0.530
Trogoniformes	Telluraves	41	9.463	0.500	0.200	0.963	0.883	0.877	0.890
Bucerotiformes	Telluraves	66	4.651	0.489	0.181	0.959	0.818	0.814	0.828
Coraciiformes	Telluraves	149	17.618	0.676	0.343	0.983	0.999	0.998	1.000
Piciformes	Telluraves	390	15.366	0.605	0.226	0.978	1.000	0.999	1.000

Strigiformes	Telluraves	76	2.241	0.523	0.207	0.962	1.000	0.999	1.000
Accipitriformes	Telluraves	240	5.044	0.482	0.169	0.961	0.976	0.972	0.980
Falconiformes	Telluraves	63	12.521	0.523	0.206	0.964	0.840	0.834	0.848
Psittaciformes	Telluraves	341	12.975	0.648	0.275	0.982	0.999	0.999	1.000
Passeriformes	Telluraves	5229	11.642	0.634	0.274	0.980	1.000	1.000	1.000
Corvides	Passeriformes	609	16.885	0.303	0.081	0.646	0.736	0.718	0.737
Meliphagoidea	Passeriformes	214	19.933	0.392	0.139	0.886	0.657	0.649	0.668
Muscicapida	Passeriformes	702	20.928	0.532	0.285	0.844	0.827	0.825	0.839
Passerida	Passeriformes	1492	2.716	0.169	0.081	0.318	0.976	0.972	0.982
Suboscines	Passeriformes	978	15.373	0.647	0.282	0.957	0.886	0.880	0.891
Sylviida	Passeriformes	1131	16.915	0.676	0.197	0.983	0.604	0.597	0.613
Aegithaloidea	Passeriformes	105	21.519	0.319	0.128	0.905	0.420	0.416	0.433
Bombycilloidea	Passeriformes	112	13.610	0.557	0.246	0.920	0.881	0.871	0.884
Cisticolidae	Passeriformes	141	13.291	0.587	0.232	0.969	0.633	0.619	0.637
Corvoidea	Passeriformes	248	3.004	0.696	0.531	0.873	1.000	1.000	1.000
Emberizoidea	Passeriformes	752	10.155	0.740	0.400	0.982	0.982	0.978	0.984
Eurylaimides	Passeriformes	51	20.351	0.290	0.170	0.493	0.902	0.901	0.913
Fringillidae	Passeriformes	87	3.692	0.205	0.108	0.494	0.719	0.712	0.727
Furnariida	Passeriformes	488	10.118	0.550	0.324	0.795	0.931	0.932	0.941
Hirundinidae	Passeriformes	77	12.973	0.818	0.513	0.989	0.972	0.968	0.974
Locustelloidea	Passeriformes	106	23.818	0.464	0.192	0.906	0.666	0.661	0.676
Malaconotoidea	Passeriformes	205	15.755	0.778	0.525	0.988	0.993	0.991	0.995
Meliphagoidea	Passeriformes	214	19.933	0.398	0.143	0.901	0.665	0.648	0.665
Motacillidae	Passeriformes	58	15.907	0.662	0.213	0.976	0.756	0.737	0.751
Muscicapoidea	Passeriformes	576	16.303	0.532	0.314	0.793	0.952	0.947	0.955
Nectariniidae	Passeriformes	166	20.611	0.386	0.223	0.646	0.944	0.943	0.953
Orioloidea	Passeriformes	54	21.037	0.664	0.202	0.981	0.706	0.692	0.709
Paridae	Passeriformes	69	11.086	0.786	0.437	0.986	0.976	0.972	0.979
Passeridae	Passeriformes	39	19.579	0.593	0.188	0.981	0.464	0.456	0.472
Petroicidae	Passeriformes	35	36.283	0.460	0.266	0.870	0.665	0.658	0.675
PloceidaeEstrildidae	Passeriformes	261	5.654	0.752	0.494	0.981	0.998	0.996	0.998

Pycnonotidae	Passeriformes	123	15.571	0.235	0.123	0.467	0.680	0.674	0.691
Sylvioidea	Passeriformes	403	13.708	0.470	0.179	0.944	0.609	0.606	0.624
Tyrannida	Passeriformes	439	25.705	0.664	0.340	0.975	0.744	0.737	0.751
Orioloidea	Corvides	54	21.037	0.486	0.161	0.956	0.536	0.531	0.549
Corvoidea	Corvides	248	3.004	0.445	0.164	0.727	0.997	0.995	0.998
Malaconotoidea	Corvides	205	15.755	0.563	0.295	0.860	0.942	0.939	0.950
Bombycilloidea	Muscicapida	112	13.610	0.327	0.129	0.830	0.608	0.600	0.617
Muscicapoidea	Muscicapida	576	16.303	0.276	0.096	0.835	0.593	0.592	0.610
Nectariniidae	Passerida	166	20.611	0.439	0.271	0.691	0.974	0.969	0.976
PloceidaeEstrildidae	Passerida	261	5.654	0.650	0.437	0.889	0.994	0.993	0.997
Emberizoidea	Passerida	752	10.155	0.864	0.569	0.994	0.992	0.991	0.994
Motacillidae	Passerida	58	15.907	0.753	0.254	0.987	0.812	0.803	0.817
Passeridae	Passerida	39	19.579	0.621	0.203	0.984	0.483	0.472	0.489
Eurylaimides	Suboscines	51	20.351	0.487	0.216	0.913	0.977	0.975	0.982
Tyrannida	Suboscines	439	25.705	0.437	0.141	0.951	0.491	0.482	0.501
Furnariida	Suboscines	488	10.118	0.335	0.151	0.818	0.712	0.706	0.722
Paridae	Sylviida	69	11.086	0.511	0.161	0.974	0.814	0.804	0.819
Locustelloidea	Sylviida	106	23.818	0.628	0.201	0.979	0.764	0.754	0.771
Aegithaloidea	Sylviida	105	21.519	0.674	0.218	0.982	0.706	0.704	0.719
Hirundinidae	Sylviida	77	12.973	0.555	0.276	0.973	0.881	0.873	0.887
Pycnonotidae	Sylviida	123	15.571	0.665	0.188	0.985	0.944	0.941	0.950
Sylvioidea	Sylviida	403	13.708	0.593	0.180	0.978	0.693	0.680	0.697
Cisticolidae	Sylviida	141	13.291	0.596	0.176	0.981	0.601	0.590	0.607
Fringillidae	Sylviida	87	3.692	0.658	0.183	0.985	0.967	0.959	0.967

2.3 Elaboration and innovation through time

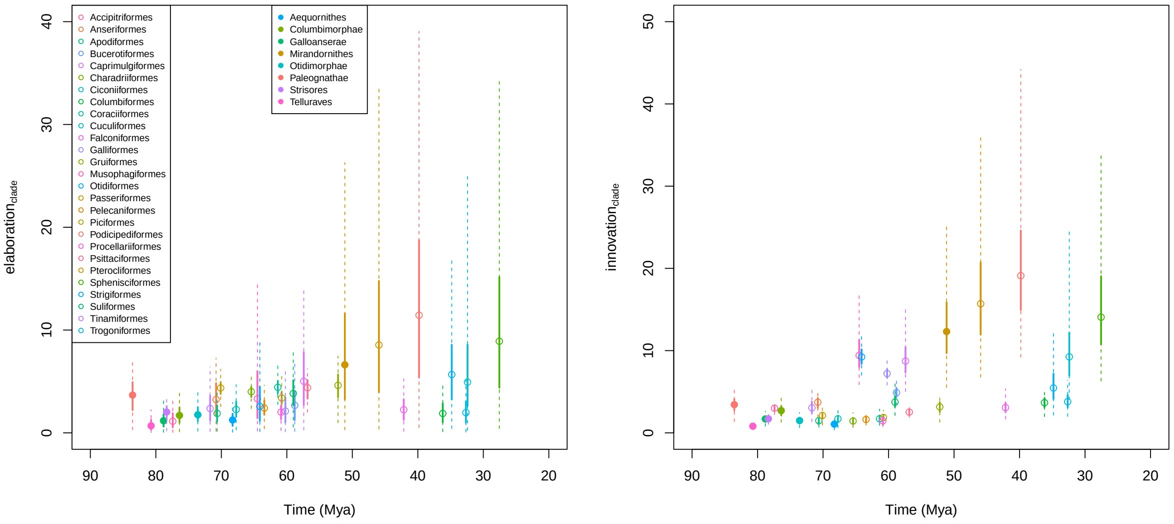

Figure S9: Distribution of $elaboration_{clade}$ and $innovation_{clade}$ for each order and super-order through time in millions of years (Mya).

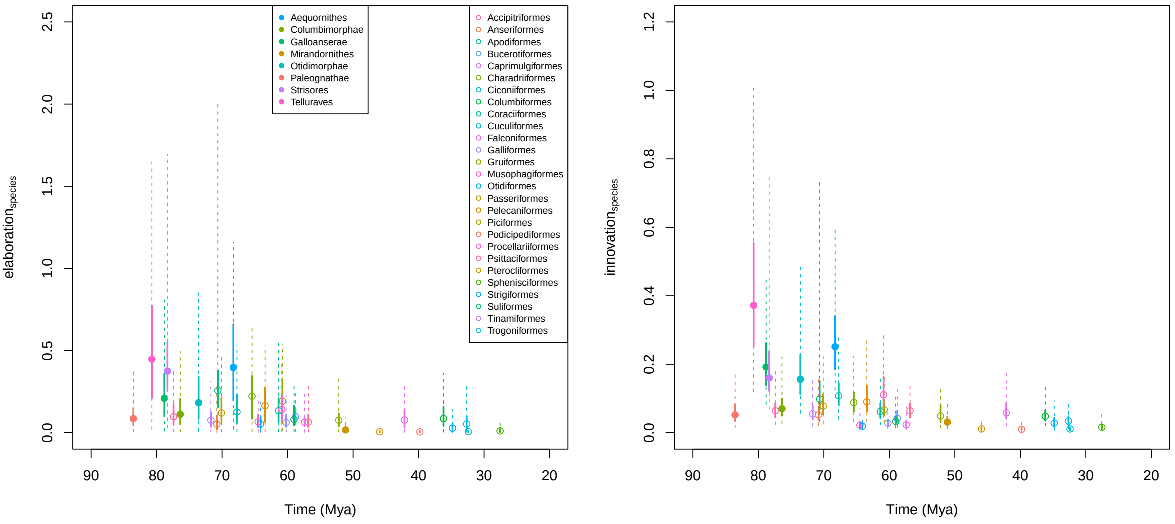

Figure S10: Distribution of $elaboration_{species}$ and $innovation_{species}$ for each order and super-order through time in millions of years (Mya).

2.4 Orientations in $elaboration_{clade}$ and $innovation_{clade}$

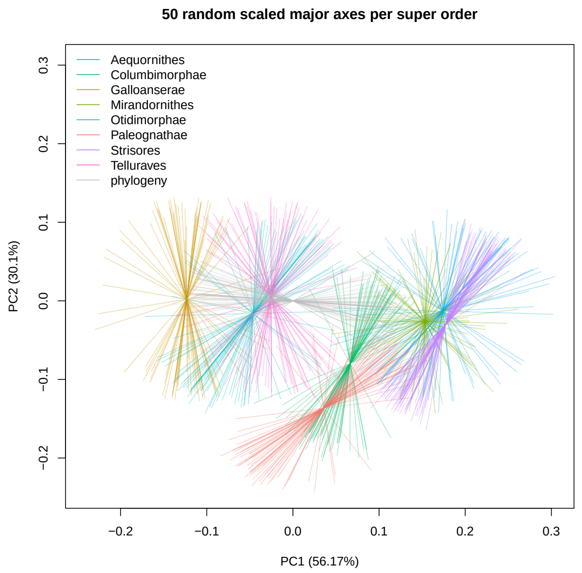

Figure S11: Distribution of 50 random scaled major axes (scaled evolutionary rate matrices eigenvectors) for each super-order illustrating that individual phylogenetic major axes of beak variation can span many different directions and that some clades have a higher spread of directions than others.

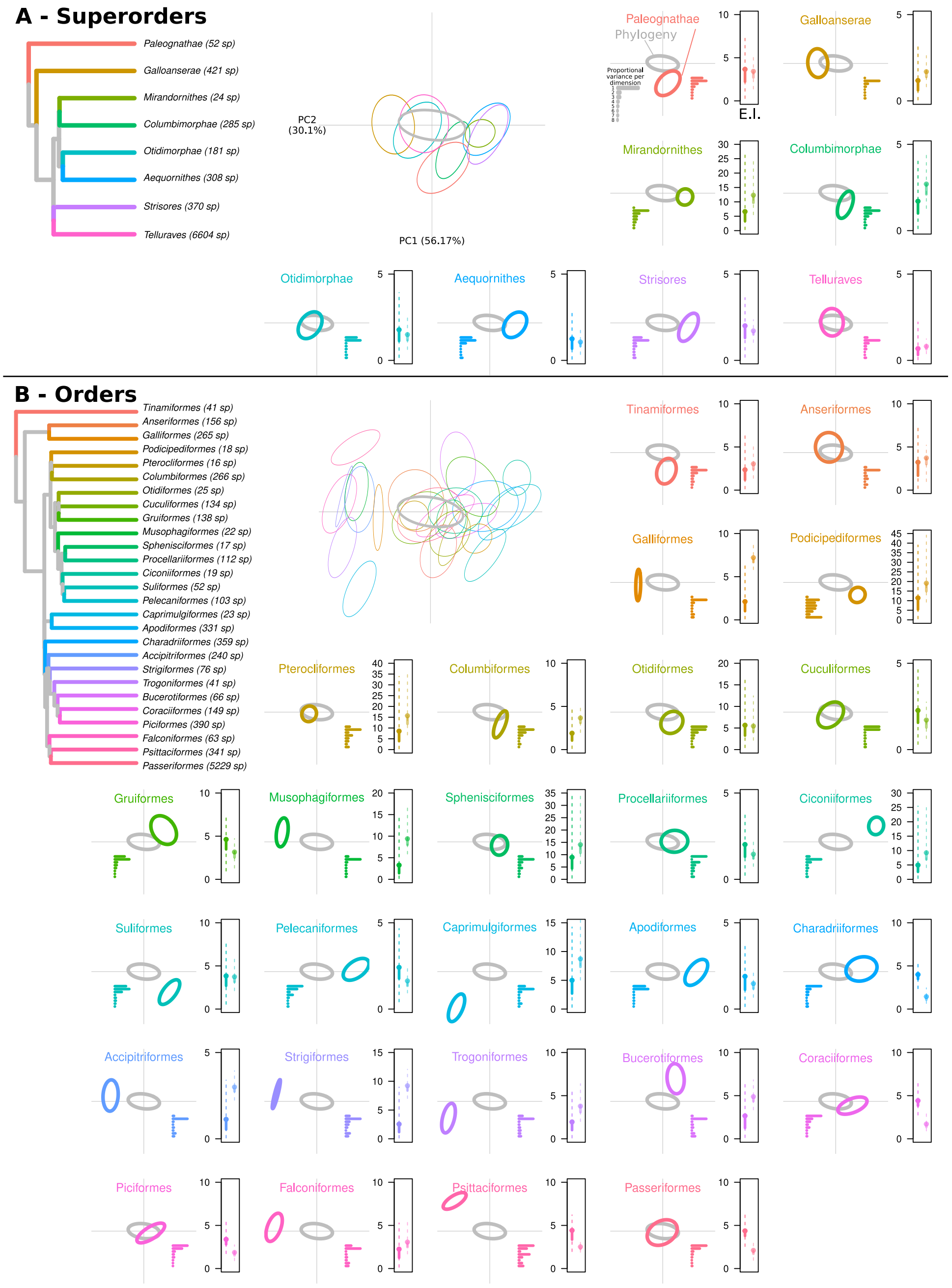

Figure S12: **(A)** Ellipses representing the scaled average posterior evolutionary rate matrix from the pGLMM models for each super-order (coloured ellipses) compared to the clade-wide phylogenetic component of the models (grey ellipses). We scaled the ellipses so the length of the phylogenetic major axis of beak variation of the clade ellipse is the same length as that of the clade-wide phylogenetic major axis ellipse (in eight dimensions). The first inset ellipse plot shows the positions of all super-order ellipses relative to the clade-wide phylogenetic ellipse. Subsequent inset plots show the results for each super-order. Inset bar plots show the proportion of variance associated with each of the eight principal component (PC) axes in shapespace. The inset boxplots correspond to the $elaboration_{clade}$ (E) and $innovation_{clade}$ (I) scores for all 4000 posterior samples. The dots represent the median $elaboration_{clade}$ and $innovation_{clade}$ values while the thick and dashed lines represent the 50% and 95% confidence intervals respectively. These scores were calculated on the unscaled ellipses resulting in different scales of $elaboration_{clade}$ and $innovation_{clade}$ for each plot. **(B)** Results for each order.

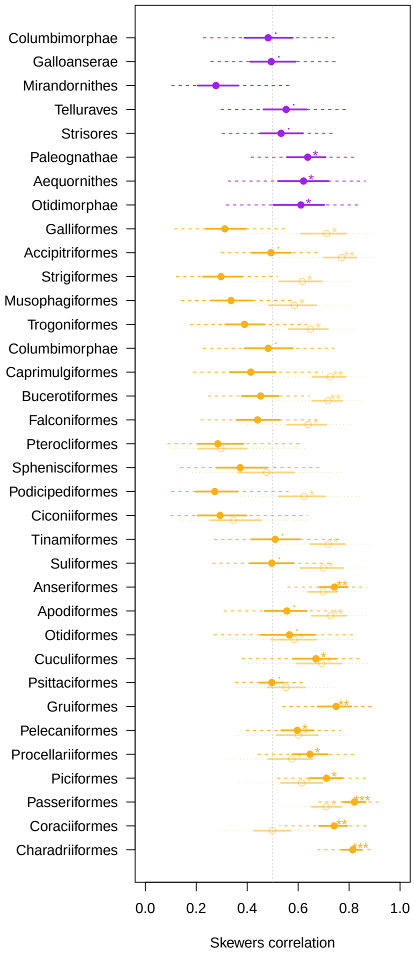

Figure S13: Random skewers test for the differences between the super-orders (in purple) and the orders (in gold) evolutionary rate matrices against the clade-wide matrices. And, in light gold, between the orders and their super-orders evolutionary rate matrices. The different line types (dashed and full) represent respectively the 95% and the 75% CI distribution and the dots represent the median skewer correlation estimate. The stars denote the median p-value estimate (0 '***' 0.001 '**' 0.01 '*' 0.05 '.' 0.1 ' ' 1).

3 Passeriformes results

3.1 Passeriformes *elaboration*$_{species}$ and *innovation*$_{species}$

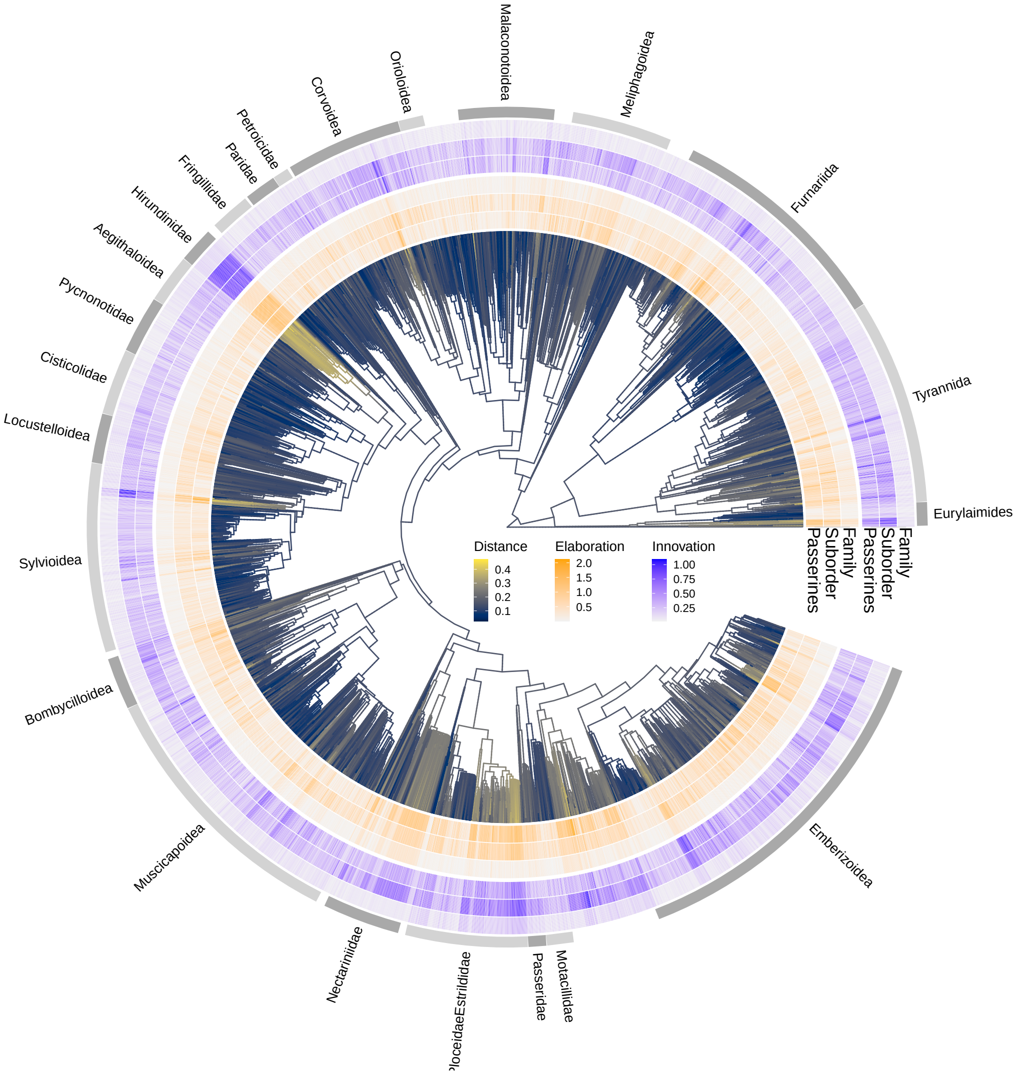

Figure S14: Passeriformes phylogeny (n = 5229 species) showing Euclidean distance of species to the centroid of beak space (branches, cividis scale), and distributions of species beak shape *elaboration*$_{species}$ (inner circle, orange scale), and *innovation*$_{species}$ (outer circle, blue scale). Elaboration and innovation scores represent comparisons of species at the order, sub-order and family level.

3.2 Passeriformes $elaboration_{clade}$ and $innovation_{clade}$ at the megaevolutionary scale

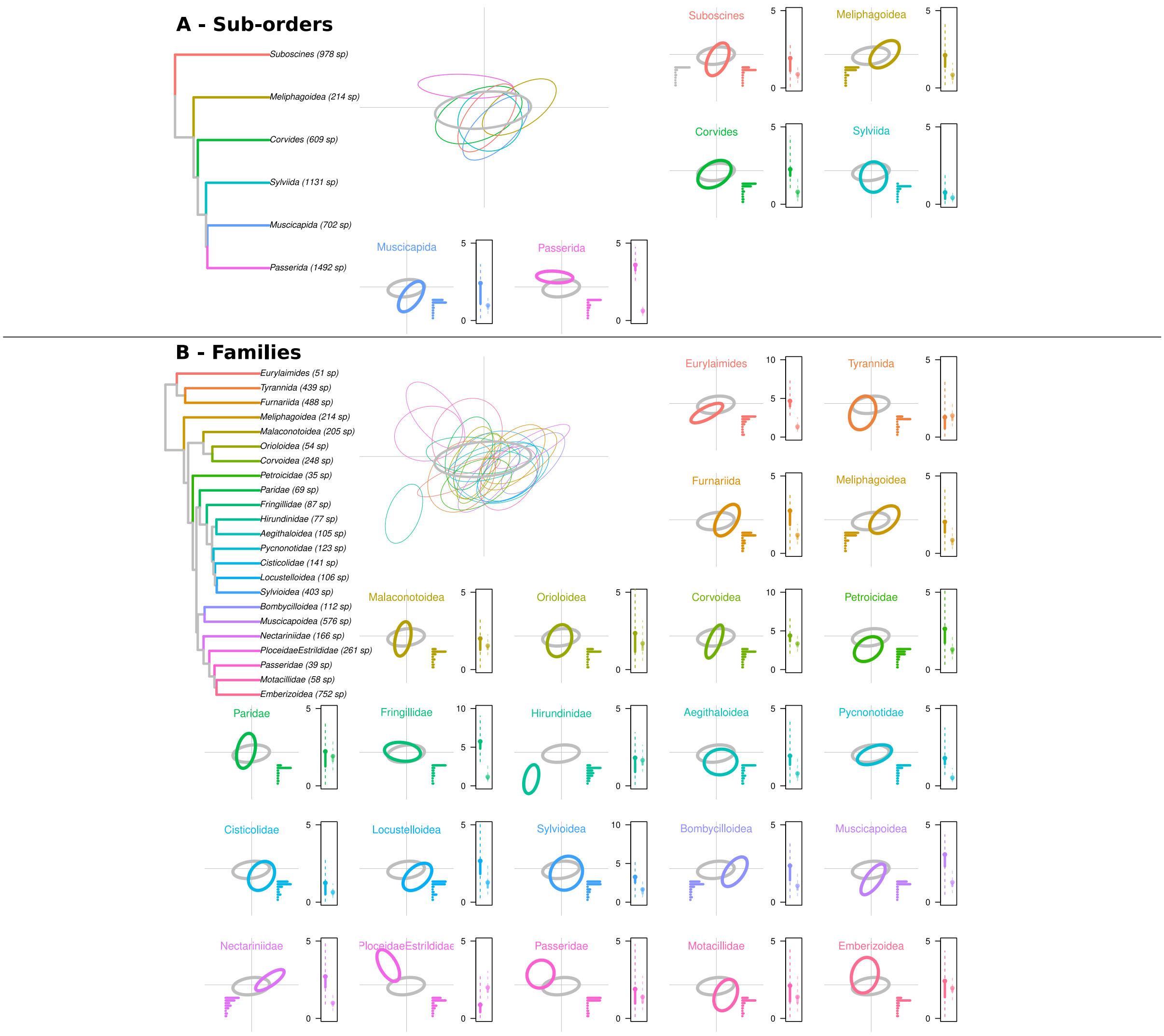

Figure S15: **(A)** Ellipses representing the scaled average posterior evolutionary rate matrix from the pGLMM models for each Passeriformes sub-orders and families (coloured ellipses) compared to the Passeriformes phylogenetic component of the models (grey ellipses). We scaled the ellipses so the length of the phylogenetic major axis of beak variation of the clade ellipse is the same length as that of the Passeriformes phylogenetic major axis ellipse (in eight dimensions). The first inset ellipse plot shows the positions of all sub-order ellipses relative to the Passeriformes phylogenetic ellipse. Subsequent inset plots show the results for each sub-order. Inset bar plots show the proportion of variance associated with each of the eight principal component (PC) axes in shapespace. The inset boxplots correspond to the $elaboration_{clade}$ (left) and $innovation_{clade}$ (right) scores for all 4000 posterior samples. The dots represent the median $elaboration_{clade}$ and $innovation_{clade}$ values while the thick and dashed lines represent the 50% and 95% confidence intervals respectively. These scores were calculated on the unscaled ellipses resulting in different scales of $elaboration_{clade}$ and $innovation_{clade}$ for each plot. **(B)** Results for each families.

3.3 Passeriformes orthogonality

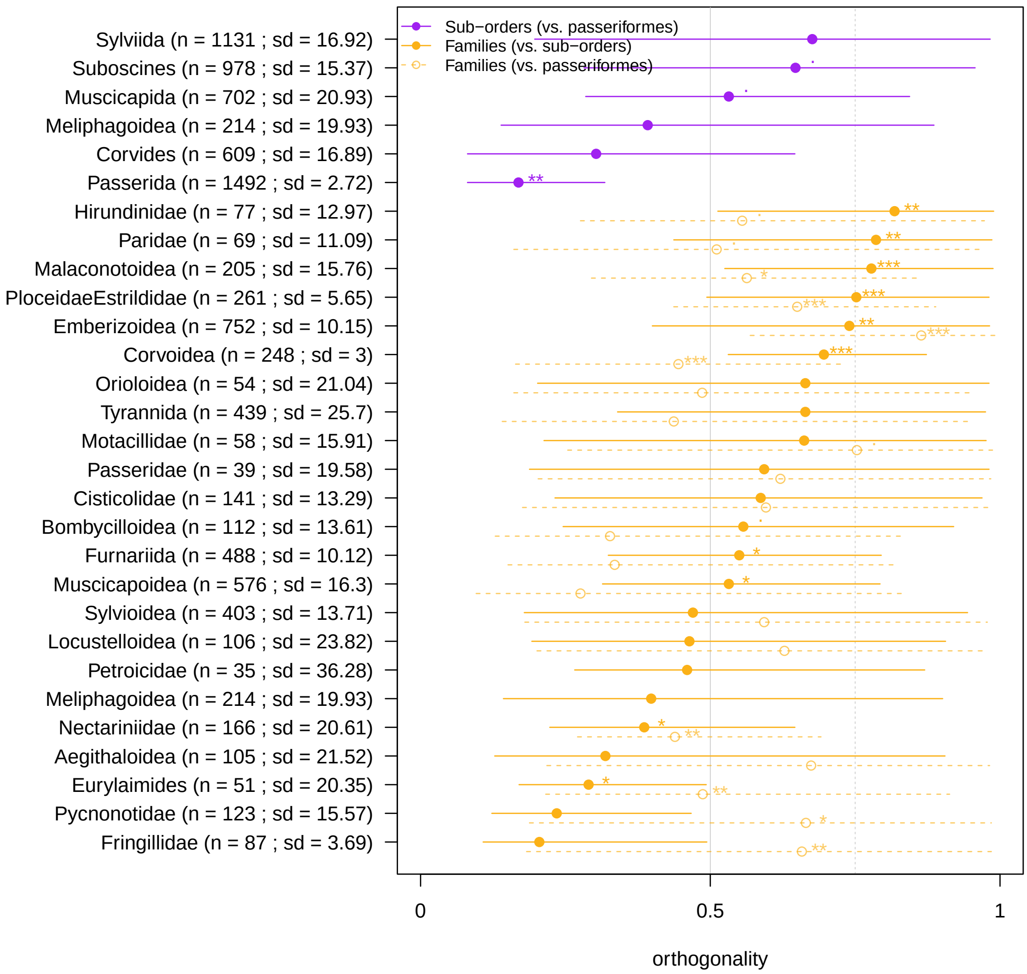

Figure S16: Amount of orthogonality of each Passeriformes sub-order's and family's phylogenetic major axis of beak variation compared to their parent or parent's parent clade. The amount of orthogonality is represented on the horizontal axis and scales from 0 (modulo of 0°) to 1 (modulo of 90°) with the background gray and dashed gray lines representing, respectively, an orthogonality of 0.5 (modulo of 45°) and 0.75 (modulo of 67.5°). Dots represent the median orthogonality of each clade and the lines their 95% confidence intervals (CI). We also indicate the number of species (n) and the standard deviation (sd) of the orientation of their phylogenetic major axis of beak variation over the 4000 variance-covariance posteriors (sd; expressed in degrees). For each clade we also measured the posterior probability of each clade's orientation being different from their parent's clade or the class-wide phylogenetic major axis of beak variation relative to their sample size and sd. The stars represent the posterior probability of the clade's orientation being different from the comparison clade (*** = pp > 0.99; ** = pp > 0.95; * = pp > 0.9; . = pp > 0.8).

4 Detailed projection and rejection operations for measuring elaboration and innovation

The following section contains the detailed definition and procedure of how we measured elaboration and innovation.

We can define the major axis from the variance-covariance matrices and then project and reject each element of interest in the space onto this axis. The following steps are generalised to n dimensions and detailed below (as well as the algorithm used in `dispRity` [64]to perform the transformations):

4.1 Defining the major axis

For O_n, the unit hypersphere matrix of n dimensions and a radius composed of the two identity matrices I_n and $-I_n$ so that:

$$O_n = \begin{pmatrix} 1 & 0 & \cdots & 0 \\ 0 & 1 & \cdots & 0 \\ \vdots & \vdots & \ddots & \vdots \\ 0 & 0 & \cdots & 1 \\ -1 & 0 & \cdots & 0 \\ 0 & -1 & \cdots & 0 \\ \vdots & \vdots & \ddots & \vdots \\ 0 & 0 & \cdots & -1 \end{pmatrix} \tag{1}$$

In other words, O_n is the matrix representing the hypersphere of n dimensions and of radius 1 that fits in the centre of the trait-space;

And O'_n is the scaled matrix hypersphere to the 95% confidence interval size using the χ^2 distribution:

$$O'_n = O_n \sqrt{\chi^2(0.95)}$$

Then, for the variance-covariance matrix VCV_n of n dimensions obtained from the posterior distribution of the `mcmcmcglmmmm`:

$$VCV_n = \begin{pmatrix} \sigma(a) & \sigma(a,b) & \cdots & \sigma(a,n) \\ \sigma(a,b) & \sigma(b) & \cdots & \sigma(b,n) \\ \vdots & \vdots & \ddots & \vdots \\ \sigma(n,a) & \sigma(n,b) & \cdots & \sigma(n) \end{pmatrix} \tag{2}$$

and the eigenvectors $\mathbf{v}$ and the eigenvalues λ satisfying the following eigen decomposition:

$$VCV_n \mathbf{v} = \lambda \mathbf{v}$$

We can get M_n, the matrix containing all the edge coordinates of the 0.95 CI hypersphere from VCV_n using the transposition of the cross product between the eigenvectors $\mathbf{v}$ and the product of the scaled 0.95 CI unit sphere O'_n and the eigenvalues λ:

$$M_n = [(O'_n \sqrt{\lambda}) \times v]^{\mathrm{T}}$$

Finally, we can centre the matrix M_n on the estimated solution of each GLMM (`model$Sol`, in `MCMCglmm` [48]) corresponding to the estimation of the position of the variance-covariance matrix in the trait-space. M_n then contains all the major axes of the 0.95 hyper-ellipse fitting the variance-covariance matrix. We can then define the first row of the matrix, $M_{1,n}$, as the major axis, the second row, $M_{2,n}$, as the second major axis (the minor axis in a 2D ellipse), etc.

The detailed procedure was adapted from Zheyuan Li's post on Stack Overflow and implemented in `dispRity::axis.covar`.

The use of the scaled 95% confidence interval hyper-sphere matrix O'_n allows both (1) to standardise the resulting M_n (the scaled version of VCV_n) so that the unit vector of the resulting space corresponds to the 95% CI on every dimension (for any dimension i in the matrix $M_{i,n}$, any vector with a norm smaller or greater than 1 is respectively inside or outside the 95% CI boundary). And (2) greatly speed up the calculations of projection and rejection in an algorithm (see below) since the hypersphere O'_n can be calculated only once for any number of matrix and the projection of rejection values are directly columns in the output transformed matrix (after the projection procedure, see below). This greatly reduces RAM and CPU usage and scaling for very large number of matrices.

4.2 Measuring projection and rejection

Once we have defined a major axis, we can project any elements in the trait-space onto that axis. Specifically, for any elements in the trait-space, we can define it as the vector $\vec{a}$ with one set of coordinates in n dimensions:

$$\vec{a} = \begin{bmatrix} x \\ y \\ \cdots \\ n \end{bmatrix} \tag{3}$$

And for any major axis that we can define as a vector $\vec{b}$ as a set of pairs of coordinates in n dimensions:

$$\vec{b} = \begin{bmatrix} x_1 & x_2 \\ y_1 & y_2 \\ \cdots & \cdots \\ n_1 & n_2 \end{bmatrix} \tag{4}$$

We can then calculate $\vec{a_1}$, the orthogonal projection of $\vec{a}$ onto $\vec{b}$ using:

$$\vec{a_1} = \frac{\vec{a} \cdot \vec{b}}{||\vec{b}||} \tag{5}$$

$$\vec{a_1} = \frac{\vec{a} \cdot \vec{b}}{||\sqrt{\vec{b} \cdot \vec{b}}||} \tag{6}$$

With $||\vec{b}|| = \sqrt{\vec{b} \cdot \vec{b}}$ being the norm of $\vec{b}$. And $\vec{a_2}$, the rejection of $\vec{a}$ onto $\vec{b}$:

$$\vec{a_2} = \vec{a} - \vec{a_1} \tag{7}$$

4.2.1 Generalisation of projection onto any vector in a set space

Using this, we can generalise the procedure so as to calculate the projection and rejection for any element within a trait-space $TS_{m,n}$:

$$TS_{m,n} = \begin{bmatrix} x_1 & x_2 & \cdots & x_m \\ y_1 & y_2 & \cdots & y_m \\ \vdots & \vdots & \ddots & \vdots \\ n_1 & n_2 & \cdots & n_m \end{bmatrix} \tag{8}$$

And any major axis defined as a vector $\vec{B}$:

$$B = \begin{bmatrix} x_1 & x_2 \\ y_1 & y_2 \\ \vdots & \vdots \\ n_1 & n_2 \end{bmatrix} \tag{9}$$

By using the linear transformation $f_{\vec{B}}$ of the trait-space TS moving $\vec{B}$ onto TS's first axis unit vector $\vec{\imath}$:

$$f_{\vec{B}}(TS) = \left(\frac{TS - [Bx_1, By_1, \cdots, Bn_1]^{\mathrm{T}}}{||\vec{B}||} \right) \cdot R_{\vec{B}}$$

With $R_{\vec{B}}$ being the rotation matrix of the vector $\vec{B}$ onto $\vec{\imath}$:

$$R_{\vec{B}} = I_{\vec{B}} - \vec{B}\vec{B}^{\mathrm{T}} - \vec{\imath}\vec{\imath}^{\mathrm{T}} + [\vec{B}\vec{\imath}] \begin{bmatrix} cos(\theta) & -sin(\theta) \\ sin(\theta) & cos(\theta) \end{bmatrix} [\vec{B}\vec{\imath}]^{\mathrm{T}} \tag{10}$$

Where θ is:

$$\theta = acos \left(\frac{\vec{B} \cdot \vec{\imath}}{||\vec{B}|| \cdot ||\vec{\imath}||} \right) \tag{11}$$

Or $\theta = acos(B_x)$ since both $||\vec{B}||$ and $||\vec{\imath}||$ are equal to 1 and $||\vec{\imath}||$ is the unit vector on the first axis.

4.3 Algorithm for calculating the projection/rejection of any element in a defined space

In practice we followed this procedure and applied a modification of this implementation (see [65]for the formal generalisation of this algorithm in n dimensions) using the following algorithm implemented in `dispRity::projections`:

1. In the trait-space, define $\vec{B}$ as the base vector (typically $\vec{B}$ is defined as the pair of coordinates from the major axis described above).
2. Centre the trait-space on the origin of $\vec{B}$ so that the first set of coordinates of $\vec{B}$ are 0.
3. Scale the trait-space to the norm of $\vec{B}$ so that the norm of $\vec{B}$ is now 1.
4. Rotate the trait-space using the rotation matrix $R_{\vec{B}}$ to satisfy the linear transformation $\vec{B} \rightarrow \vec{\imath}$ (with $\vec{\imath}$ being the first unit vector of the trait-space - typically the x axis unit vector).
5. Project/reject every element in the trait-space on $\vec{B}$ (that is now $\vec{\imath}$). In practice, the first coordinate (x) of each element is now its projection onto $\vec{B}$.

4.4 Definition of species and clade innovation and elaboration

From the definitions above, we can define $\text{elaboration}_{\text{species}}$ and $\text{innovation}_{\text{species}}$, the elaboration and innovation at the macroevolutionary (species level) and $\text{elaboration}_{\text{clade}}$ and $\text{innovation}_{\text{clade}}$, the elaboration and innovation at the megaevolutionary level (clade).

For any species with coordinates $\vec{species}$ in a n dimensional space, we can estimate VCV_{clade}, the variance-covariance matrices representing the clade that contains $\vec{species}$. We can then calculate the eigen vector and eigenvalues from these matrices as:

$\mathbf{v}_{clade}$ defined as:

$$VCV_{clade}\mathbf{v}_{clade} = \lambda_{clade}\mathbf{v}_{clade} \tag{12}$$

In practice, these are solved using a eigen decomposition in R `base::eigen`.

We can then define the followings for any species x:

$$\text{elaboration}_{\text{species}_x} = ||\frac{\vec{species}_x \cdot \mathbf{v}_{clade}}{\sqrt{\mathbf{v}_{clade} \cdot \mathbf{v}_{clade}}}|| \tag{13}$$

With $\vec{species}_x$ being the vector in n dimensional space for species x. And $\mathbf{v}_{clade}$ being the eigen vector of a specific clade (e.g. $\mathbf{v}_{clade_c}$)

$$\text{innovation}_{\text{species}_x} = \|\vec{species}_x - \text{elaboration}_{\text{species}_x}\| \tag{14}$$

And the followings for any clade c (withing a parent clade $c - 1$):

$$\text{elaboration}_{\text{clade}_c} = \|\frac{\mathbf{v}_{clade_c} \cdot \mathbf{v}_{clade_{c-1}}}{\sqrt{\mathbf{v}_{clade_{c-1}} \cdot \mathbf{v}_{clade_{c-1}}}}\| \tag{15}$$

$$\text{innovation}_{\text{clade}_c} = \|\mathbf{v}_{clade_c} - \text{elaboration}_{\text{clade}_c}\| \tag{16}$$

4.5 Relation between elaboration and innovation and metrics in micro/macro-evolution

4.5.1 Relative eigenvalues dispersion

[44, 66] propose to describe a variance-covariance matrix in terms of its eigen dispersion V_{rel} as:

$$V_{rel} = \frac{\sum_i^n (\lambda_i - \sum \frac{\lambda_i}{n})^2}{n(n-1) \sum \frac{\lambda_i}{n}^2} \tag{17}$$

Which is the dispersion of eigenvalues (squared difference between each eigen value and the average eigen value) where λ_i are each individual eigen values and n is the number of dimensions considered.

Using this definition and the definitions above of $\text{elaboration}_{\text{clade}_c}$ and $\text{innovation}_{\text{clade}_c}$ we can calculate the and plot the relation between elaboration and innovation and their relative eigen dispersion:

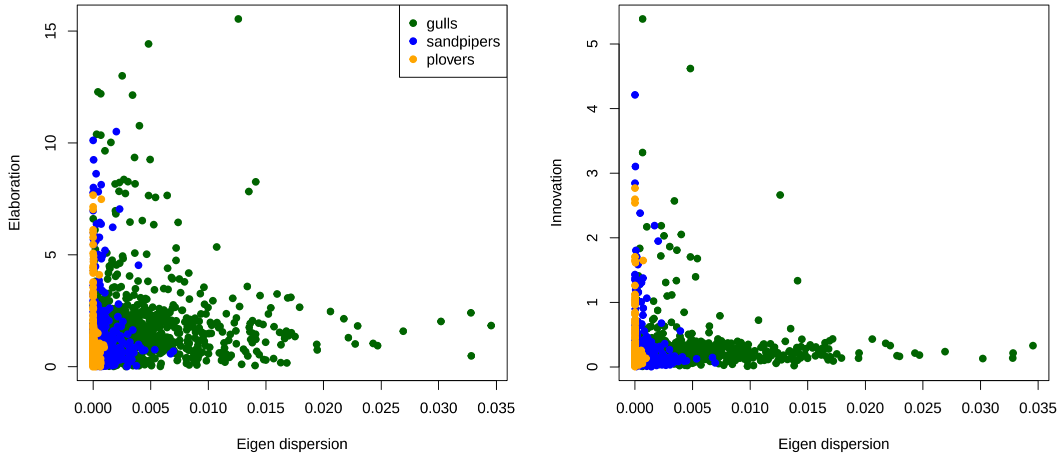

Figure S17: Relation between relative eigen dispersion [44, 66] and elaboration$_{\text{clade}_c}$ and innovation$_{\text{clade}_c}$ on the `charadriiformes` example dataset from the `dispRity` package.

4.5.2 Evolvability and respondability

[42] propose to measure the evolvability and respondability of an individual as their "response to selection in n traits". Here, some equivalent can be drawn (albeit cautiously) between the response of an individual or a group (population) to selection pressure and a species. Arguably, a species is the end product of this (and many other) selection pressures on a group of individuals. If so, then, we can relate evolvability and respondability [42] for a $species_x$ to it's elaboration$_{\text{species}_x}$ and innovation$_{\text{species}_x}$ as follows:

$$\text{evolvability}_{\text{species}_x} = \text{elaboration}_{\text{species}_x} \tag{18}$$

and, using Pythagorean theorem in a right angled triangle:

$$\text{respondability}_{\text{species}_x} = \sqrt{\text{elaboration}^2_{\text{species}_x} + \text{innovation}^2_{\text{species}_x}} \tag{19}$$

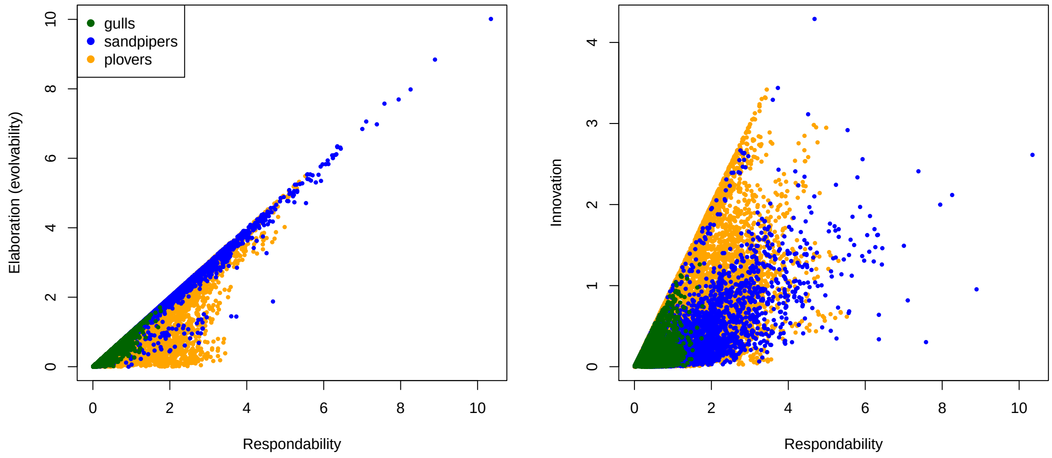

Figure S18: Relation between respondability [42] and elaboration$_{\text{species}}$ and innovation$_{\text{species}}$ on the `charadriiformes` example dataset from the `dispRity` package.

Conditional evolvability however will be more complex to determine since it is defined as the relation between evolvability and autonomy which is specific defined in [42] as the inverse of the **G** matrix. In [42] , the inverse of **G** is defined as "the fraction of the genetic variation that is independent of potentially constraining characters" which in our case will be harder to define. Although it would technically be possible to use the inverse of the **R** matrix used here (VCV_{clade}), it is unclear what it would represent biologically.

REFERENCES AND NOTES

1. Multiple routes to mammalian diversity. C. Venditti, A. Meade, M. Pagel, *Nature* **479**, 393–396 (2011).
2. Fast and accurate detection of evolutionary shifts in Ornstein–Uhlenbeck models. M. Khabbazian, R. Kriebel, K. Rohe, C. Ane, *Methods Ecol. Evol.* **7**, 811–824 (2016).
3. S. Castiglione, G. Tesone, M. Piccolo, M. Melchionna, A. Mondanaro, C. Serio, M. di Febbraro, P. Raia, A new method for testing evolutionary rate variation and shifts in phenotypic evolution. *Methods Ecol. Evol.* **9**, 974–983 (2018).
4. T. F. Hansen, G. H. Bolstad, M. Tsuboi, Analyzing disparity and rates of morphological evolution with model-based phylogenetic comparative methods. *Syst. Biol.* **71**, 1054–1072 (2021).
5. O. Larouche, S. M. Gartner, M. W. Westneat, K. M. Evans, Mosaic evolution of the skull in labrid fishes involves differences in both tempo and mode of morphological change. *Syst. Biol.* **72**, 419–432 (2022).
6. M. Pagel, C. O'Donovan, A. Meade, General statistical model shows that macroevolutionary patterns and processes are consistent with Darwinian gradualism. *Nat. Commun.* **13**, 1113 (2022).
7. G. Navalón, A. Bjarnason, E. Griffiths, R. B. Benson, Environmental signal in the evolutionary diversification of bird skeletons. *Nature* 306–311 (2022).
8. D. N. Basu, V. Bhaumik, K. Kunte, The tempo and mode of character evolution in the assembly of mimetic communities. *Proc. Natl. Acad. Sci. U.S.A.* **120**, e2203724120 (2023).
9. C. R. Cooney, J. A. Bright, E. J. R. Capp, A. M. Chira, E. C. Hughes, C. J. A. Moody, L. O. Nouri, Z. K. Varley, G. H. Thomas, Mega-evolutionary dynamics of the adaptive radiation of birds. *Nature* **542**, 344–347 (2017).
10. J. B. Smaers, R. S. Rothman, D. R. Hudson, A. M. Balanoff, B. Beatty, D. K. N. Dechmann, D. de Vries, J. C. Dunn, J. G. Fleagle, C. C. Gilbert, A. Goswami, A. N. Iwaniuk, W. L. Jungers, M. Kerney, D. T. Ksepka, P. R. Manger, C. S. Mongle, F. J. Rohlf, N. A. Smith, C. Soligo, V. Weisbecker, K. Safi, The evolution of mammalian brain size. *Sci. Adv.* **7**, eabe2101 (2021).
11. A. Goswami, E. Noirault, E. J. Coombs, J. Clavel, A. C. Fabre, T. J. D. Halliday, M. Churchill, A. Curtis, A. Watanabe, N. B. Simmons, B. L. Beatty, J. H. Geisler, D. L. Fox, R. N. Felice, Attenuated evolution of mammals through the Cenozoic. *Science* **378**, 377–383 (2022).
12. G. G. Simpson, *Tempo and mode in evolution* (Columbia University Press, 1984).
13. G. G. Simpson, *The major features of evolution* (Columbia University Press, 1953).
14. D. Schluter, Adaptive radiation along genetic lines of least resistance. *Evolution* **50**, 1766–1774 (1996).

15. G. Marroig, J. M. Cheverud, Size as a line of least evolutionary resistance: Diet and adaptive morphological radiation in New World monkeys. *Evolution* **59**, 1128–1142 (2005).
16. T. Hansen, *Encyclopedia of Evolutionary Biology*, R. M. Kliman, ed. (Academic Press, 2016), pp. 83–89.
17. D. Rhoda, A. Haber, K. D. Angielczyk, bioRxiv 2022.08.12.503188 (2022). https://doi.org/10.1101/2022.08.12.503188
18. D. Jablonski, Evolvability and macroevolution: Overview and synthesis. *Evol. Biol.* **49**, 265–291 (2022)
19. G. H. Bolstad, T. F. Hansen, C. Pélabon, M. Falahati-Anbaran, R. Pérez-Barrales, W. S. Armbruster, Genetic constraints predict evolutionary divergence inDalechampiablossoms. *Philos. Trans. R Soc B Biol. Sci.* **369**, 20130255 (2014).
20. G. M. Walter, J. D. Aguirre, M. W. Blows, D. Ortiz-Barrientos, Evolution of genetic variance during adaptive radiation. *Am. Nat.* **191**, E108–E128 (2018).
21. J. W. McGlothlin, M. E. Kobiela, H. V. Wright, D. L. Mahler, J. J. Kolbe, J. B. Losos, E. D. Brodie III, Adaptive radiation along a deeply conserved genetic line of least resistance inAnolislizards. *Evol. Lett.* **2**, 310–322 (2018)
22. M. Tsuboi, W. van der Bijl, B. T. Kopperud, J. Erritzøe, K. L. Voje, A. Kotrschal, K. E. Yopak, S. P. Collin, A. N. Iwaniuk, N. Kolm, Breakdown of brain–Body allometry and the encephalization of birds and mammals *Nat. Ecol. Evol.* **2**, 1492–1500 (2018).
23. C. S. Mongle, A. Nesbitt, F. A. Machado, J. B. Smaers, A. H. Turner, F. E. Grine, J. C. Uyeda, A common mechanism drives the alignment between the micro- and macroevolution of primate molars. *Evolution* **76**, 2975–2985 (2022).
24. G. Burin, T. Park, T. D. James, G. J. Slater, N. Cooper, The dynamic adaptive landscape of cetacean body size. *Curr. Biol.* **33**, 1787–1794.e3 (2022).
25. A. G. Jones, S. J. Arnold, R. Burger, Evolution and stability of the G-matrix on a landscape with a moving optimum. *Evolution* **58**, 1639–1654 (2004).
26. M. N. Fasanelli, P. S. Milla Carmona, I. M. Soto, D. T. Tuero, Allometry, sexual selection and evolutionary lines of least resistance shaped the evolution of exaggerated sexual traits within the genus Tyrannus. *J. Evol. Biol.*, 1–11 (2022).
27. M. W. Blows, M. Higgie, Genetic constraints on the evolution of mate recognition under natural selection. *Am. Nat.* **161**, 240–253 (2003).
28. D. Houle, G. H. Bolstad, K. van der Linde, T. F. Hansen, Mutation predicts 40 million years of fly wing evolution. *Nature* **548**, 447–450 (2017).

29. F. A. Machado, Selection and constraints in the ecomorphological adaptive evolution of the skull of living canidae (Carnivora, Mammalia). *Am. Nat.* **196**, 197–215 (2020).
30. D. S. Caetano, L. J. Harmon, ratematrix: An R package for studying evolutionary integration among several traits on phylogenetic trees. *Methods Ecol. Evol.* **8**, 1920–1927 (2017).
31. D. S. Caetano, L. J. Harmon, Estimating correlated rates of trait evolution with uncertainty. *Syst. Biol.* **68**, 412–429 (2018).
32. G. J. Slater, A. R. Friscia, Hierarchy in adaptive radiation: A case study using the Carnivora (Mammalia). *Evolution* **73**, 524–539 (2019).
33. A. M. Olsen, Feeding ecology is the primary driver of beak shape diversification in waterfowl. *Funct. Ecol.* **31**, 1985–1995 (2017)
34. R. N. Felice, J. A. Tobias, A. L. Pigot, A. Goswami, Dietary niche and the evolution of cranial morphology in birds. *Proc. R Soc. B Biol. Sci.* **286**, 20182677 (2019).
35. J. A. Bright, J. Marugán-Lobón, S. N. Cobb, E. J. Rayfield, The shapes of bird beaks are highly controlled by nondietary factors. *Proc. Natl. Acad. Sci. U.S.A.* **113**, 5352–5357 (2016).
36. G. Navalón, J. A. Bright, J. Marugán-Lobón, E. J. Rayfield, The evolutionary relationship among beak shape, mechanical advantage, and feeding ecology in modern birds. *Evolution* **73**, 422–435 (2019).
37. A. L. Pigot, C. Sheard, E. T. Miller, T. P. Bregman, B. G. Freeman, U. Roll, N. Seddon, C. H. Trisos, B. C. Weeks, J. A. Tobias, Macroevolutionary convergence connects morphological form to ecological function in birds. *Nat. Ecol. Evol.* **4**, 230–239 (2020).
38. A.-M. Chira, C. R. Cooney, J. A. Bright, E. J. R. Capp, E. C. Hughes, C. J. A. Moody, L. O. Nouri, Z. K. Varley, G. H. Thomas, Correlates of rate heterogeneity in avian ecomorphological traits. *Ecol. Lett.* **21**, 1505–1514 (2018).
39. G. Navalón, S. M. Nebreda, J. A. Bright, M. Fabbri, R. B. J. Benson, B.-A. Bhullar, J. Marugán-Lobón, E. J. Rayfield, Craniofacial development illuminates the evolution of nightbirds (Strisores). *Proc. Royal Soc. B Biol. Sci.* **288**, 20210181 (2021).
40. G. Mayr, A new Eocene swift-like bird with a peculiar feathering. *Ibis* **145**, 382–391 (2003).
41. D. T. Ksepka, J. A. Clarke, S. J. Nesbitt, F. B. Kulp, L. Grande, Fossil evidence of wing shape in a stem relative of swifts and hummingbirds (Aves, Pan-Apodiformes). *Proc. Biol. Sci.* **280**, 20130580 (2013).
42. T. F. Hansen, D. Houle, Measuring and comparing evolvability and constraint in multivariate characters. *J. Evol. Biol.* **21**, 1201–1219 (2008).

43. M. Pavlicev, J. M. Cheverud, G. P. Wagner, Measuring morphological integration using eigenvalue variance. *Evol. Biol.* **36**, 157–170 (2009).
44. J. Watanabe, Statistics of eigenvalue dispersion indices: Quantifying the magnitude of phenotypic integration. *Evolution* **76**, 4–28 (2022)
45. P. C. Phillips, S. J. Arnold, Hierarchical comparison of genetic variance-covariance matrices. I. Using the flury hierarchy. *Evolution* **53**, 1506–1515 (1999).
46. J. A. Endler, D. A. Westcott, J. R. Madden, T. Robson, Animal visual systems and the evolution of color patterns: Sensory processing illuminates signal evolution. *Evolution* **59**, 1795–1818 (2005).
47. E. C. Hughes, D. P. Edwards, J. A. Bright, E. J. R. Capp, C. R. Cooney, Z. K. Varley, G. H. Thomas, Global biogeographic patterns of avian morphological diversity. *Ecol. Lett.* **25**, 598–610 (2022).
48. J. D. Hadfield, MCMC methods for multi-response generalized linear mixed models: The MCMCglmm R package. *J. Stat. Softw.* **33**, 1–22 (2010).
49. S. J. Steppan, P. C. Phillips, D. Houle, Comparative quantitative genetics: Evolution of the G matrix. *Trends Ecol. Evol.* **17**, 320–327 (2002).
50. M. R. Robinson, A. P. Beckerman, Quantifying multivariate plasticity: Genetic variation in resource acquisition drives plasticity in resource allocation to components of life history. *Ecol. Lett.* **16**, 281–290 (2013).
51. J. A. Bright, J. Marugán-Lobón, E. J. Rayfield, S. N. Cobb, The multifactorial nature of beak and skull shape evolution in parrots and cockatoos (Psittaciformes). *BMC Evol. Biol.* **19**, 104 (2019)
52. L. S. T. Ho, C. Ané, A linear-time algorithm for Gaussian and non-Gaussian trait evolution models. *Syst. Biol.* **63**, 397–408 (2014).
53. T. F. Hansen, Stabilizing selection and the comparative analysis of adaptation. *Evolution* **51**, 1341 (1997).
54. A.-M. Chira, C. R. Cooney, J. A. Bright, E. J. R. Capp, E. C. Hughes, C. J. A. Moody, L. O. Nouri, Z. K. Varley, G. H. Thomas, The signature of competition in ecomorphological traits across the avian radiation. *Proc. R. Soc. B* **287**, 20201585 (2020).
55. R Core Team, *R: A Language and Environment for Statistical Computing*, R Foundation for Statistical Computing, Vienna, Austria (2022).
56. S. Schlager, *Statistical Shape and Deformation Analysis*, G. Zheng, S. Li, G. Szekely, eds. (Academic Press, 2017), pp. 217–256.

57. W. Jetz, G. H. Thomas, J. B. Joy, K. Hartmann, A. O. Mooers, The global diversity of birds in space and time. *Nature* **491**, 444–448 (2012).
58. E. Paradis, K. Schliep, ape 5.0: An environment for modern phylogenetics and evolutionary analyses in R. *Bioinformatics* **35**, 526–528 (2019).
59. T. Guillerme, N. Cooper, TGuillerme/mcmcmcglmmm: v0.9, Zenodo (2022); https://doi.org/10.5281/zenodo.6974582.
60. J. M. Cheverud, G. Marroig, Research article comparing covariance matrices: Random skewers method compared to the common principal components model. *Genet. Mol. Biol.* **30**, 461–469 (2007)
61. M. Pagel, Inferring evolutionary processes from phylogenies. *Zoologica Scripta* **26**, 331–348 (1997).
62. T. Guillerme, N. Cooper, A. Beckerman, G. Thomas, Innovation and elaboration on the avian tree of life. bioRxiv 2022.08.12.503188 (2022). https://doi.org/10.1101/2022.08.12.503188
63. T. Guillerme, N. Cooper, Elaboration and innovation in bird beaks. bioRxiv 2022.08.12.503188 (2022). https://doi.org/10.1101/2022.08.12.503188
64. T. Guillerme, dispRity: A modular R package for measuring disparity. *Methods Ecol. Evol.* **9**, 1755–1763 (2018)
65. A. Aguilera, R. Pérez-Aguila, General n-dimensional rotations, WSCG SHORT Communication papers proceedings (2004); https://dspace5.zcu.cz/bitstream/11025/6178/1/N29.pdf.
66. L. Van Valen, Multivariate structural statistics in natural history. *J. Theor. Biol.* **45**, 235–247 (1974).
